# Sleeplessness: A Difficult Problem in Therapeutics

**Published:** 1916-12-23

**Authors:** 


### SLEEPLESSNESS.

### A Difficult Problem in Therapeutics.

Always troublesome, and sometimes of the gravest import, sleeplessness is a condition that must be treated, and that it is easy to treat so as to do more harm than good. People differ much with respect to the amount of sleep they need. Some cannot do their work in comfort and without strain unless they have as much as nine hours' sleep out of the twenty-four. Many people require eight, and from seven to eight is the usual allowance; but there are people who never sleep for more than three or four hours, and if they get as much as this on end, and without waking, they congratulate themselves on having had a good night. Some, if disturbed in the night, wake easily, rapidly, and completely, with all their wits about them: others are roused with difficulty, and are at first dazed and confused. Some can take their sleep in snatches, at odd hours, and in almost any circumstances, and when they please: others cannot sleep except in comfort, in quiet, in the dark, and at their accustomed time. Some people sleep the whole night through, others wake many times: some sleep heavily and dream little or not at all: others sleep lightly, and habitually dream so much and so vividly that their brains seem to have no rest, but yet their sleep is not less refreshing than that of people who do not dream at all. All these idiosyncrasies must be taken into account in deciding whether a person sleeps well or badly. Everyone has his own individual standard, and if he gets as much sleep as suffices for him, we need not be disturbed that he gets less than would suffice for ourselves, or for that convenient fiction, the average or normal man. Moreover, we must remember that people who are not sleeping as well as usual are apt to underestimate the amount of sleep they get. This is especially the case with those whose sleep is discontinuous. The intervals during which they are awake seem to them long because they are tedious: the intervals during which they sleep, especially if they are dreamless, seem short because there is nothing to mark their duration.

### Drugs to be a Last Resort.

When, however, we have determined that the amount of sleep a person procures is less than what is normal for him, we must take steps to secure to him his normal amount, and of these steps the last that should be employed is the administration of hypnotic drugs. These should be reserved for the cases that will not yield to other remedies. The first measure to take is based upon the fact that there is a close connection between sleep and digestion. Observation of animals of all kinds shows that the natural time of sleep is after food, and that a heavy meal is followed by drowsiness. We see the same regular sequence in the experience of the British paterfamilias on a Sunday afternoon. On week-days he takes a light meal in the middle of the day, for he finds that after a heavy lunch he is unfit for work. If his busy surroundings prevent him from feeling actually sleepy, they do not prevent him from being lethargic and dull, or from feeling that he has not all his wits about him; so his lunch is but light, and he reserves his heavy meal until evening. But on Sundays he takes his heavy meal in the middle of the day, and he pays for it in a heavy snooze in the afternoon. The lesson is impressive, and should teach us that one of the conditions of drowsiness is a full stomach, and that no one, unless greatly fatigued, can sleep on an empty belly. It does not follow, however, that we can ensure drowsiness by administering a heavy meal. This is true of those only whose digestion is unimpaired. Dyspeptics are bad sleepers, eupeptics are good sleepers; but even those whose digestions are good will be kept awake by a meal they cannot digest, or by a meal which, though generally digestible, yet contains some ingredient, such as a half-cooked vegetable, which they cannot digest without difficulty. We shall find that very many of those who complain of sleeplessness are dyspeptic, and for them the proper measure to procure sleep is not to give hypnotic drugs but to relieve the dyspepsia. If this is done, hypnotic drugs will not be needed.

### Alimentary Supervision.

The first treatment of sleeplessness should be addressed, not to the brain, but to the stomach. It is not enough that the stomach should be full. It is necessary also that the food it contains should be such as can be digested without disturbance and without strain. It does not follow, however, that what is called "light diet," by which is usually meant milky and farinaceous puddings, is the best for this purpose. Such puddings, being of a pappy consistence, are usually swallowed without mastication; they are therefore insufficiently salivated, and are for this reason by no means easy of digestion to those whose digestions are impaired. What diet is suitable in any particular case of dyspepsia depends upon the character of the dyspepsia; but, in any case, the first step in the treatment of sleeplessness in a dyspeptic is to find a diet that the patient can digest without difficulty, and, when this is found, further steps will usually be unnecessary. In healthy people with good digestions, the heavier the meal, the greater the drowsiness that follows, and the greater the difficulty of keeping awake; but this rule does not obtain with invalids, nor with those whose digestions are impaired. With such people nothing tends more to vigilance than a heavy meal; unless, indeed, it is an empty stomach. Both are enemies to sleep.

The second natural cause of sleepiness is fatigue. It is frequent for a patient who passes the day in complete idleness to complain that he, or more often she, does not sleep. But why should she? Sleep is tired nature's sweet restorer, and if nature is not tired, how can sleep be expected?

There is here a double reason for wakefulness. Exercise not only brings healthy and sleep-producing fatigue, but also is one of the conditions of good digestion. The weary Willie or the *malade imaginaire* who spends his day on his sofa or in his easy-chair, overloading his digestive organs with over-frequent meals that he has not earned, complains that he does not sleep. He probably sleeps a great deal more than he will admit, and, if he does not, it is his own fault. He has done nothing to earn his sleep, and he does not deserve it. To dose such a patient with hypnotic drugs is immoral. Let him lie awake. If, indeed, his idleness is enforced by circumstances; if he fails to take exercise because he has broken his leg; then indeed he is to be pitied; but then he sleeps less because he needs less sleep; and, if he complains, this should be explained to him. In such cases the massage that saves him from a stiff limb performs a double function. It gives him a substitute for exercise and enables him to sleep. But if he is capable of taking exercise, let him take it, and by so doing increase in two ways his chances of a good night—first by experiencing healthy fatigue, and, second, by the improvement of digestion that exercise produces.

If, however, he is crippled, or for any reason cannot walk or use his arms strenuously, we may prescribe what is ridiculously called "carriage exercise"; with this proviso, that the carriage must be an open carriage. Carriage exercise in a closed carriage is about as efficacious as sitting in an easy-chair, which we might equally well call "easy-chair exercise." Why it should be so is not easy to conjecture, but it is an undoubted fact that facing the wind, allowing the wind to blow on the bare face, has a strong hypnotic effect, and alone is in some cases a remedy for sleeplessness. Hence, no doubt, the hypnotic effect, which is generally acknowledged, of motoring.

### Subsidiary Aids.

There are subsidiary aids to sleep that should be tried before hypnotic drugs are administered. People who live sedentary lives and employ their brains actively are apt to suffer from cold feet. George Eliot is said to have written her novels with her feet in hot water on this account. Cold feet are very inimical to sleep, and sleeplessness from this cause may be remedied by the simple means of a hot-water bottle, without any recourse to drugs. Again, heat is inimical to sleep. Sojourners in very hot climates find this the most distressing effect of the climate. On the other hand, the soporific effect of cold is notoriously one of the greatest dangers of Arctic travelling. The desire to sink to sleep in the snow is well known to be almost overpowering. This teaches us to inquire whether the patient who complains of sleeplessness does not load himself too heavily with bed-clothes. In some cases the removal of a down quilt or a blanket is a very effectual hypnotic.

If all these measures fail, and if we are assured that the patient really does sleep much less than he is accustomed, and than any change in his mode of life renders natural, we may be compelled at length to use hypnotic drugs, and of these we have now a wide choice. Within the memory of men now in practice, opium and its preparations were the only drugs available for procuring sleep, and they are still the best for that sleeplessness which is caused by the crude pain of bodily injury or disease. The first drug introduced to supplement the preparations of opium was hydrate of chloral, which, reinforced or not by bromide of potassium, held the field for many years as the sole hypnotic in cases of sleeplessness not due to pain. Now there is a profusion of hypnotic drugs, each of which has its own peculiar merit and defect. These need not be particularised; but, common to them all, and to the opium preparations as well, is the danger of establishing an uncontrollable habit by constant administration.

### The Drug Habit.

None of them, therefore, should be given habitually, nor should any be given without good cause. No one, however bad a sleeper he may be, should have an hypnotic drug by routine every night of his life. There are very few indeed, if any, who would not have an occasional good night without a drug; and there are none who can thus be treated by routine without the danger of becoming slaves to the craving for a drug. Nor is the ill-effect confined to the patient. The doctor who thus treats him is falling into a habit of intellectual laziness and inefficiency, and is allowing himself to deteriorate. He is losing his intellectual grip and interest. He is becoming a creature, not of intelligence, but of habit. Even when some hypnotic drug must be given frequently, the same drug should not be given frequently. The drug should be varied from time to time, so as to avoid the danger of establishing a craving for any one. Above all, the administration of the drug should never be left to the patient's own discretion. However frequently it may have to be given, it should never be given without the doctor's sanction for the particular individual occasion. To entrust a hypodermic syringe and a supply of morphia to a patient, with instructions to administer it to himself when necessary, ought to be made a criminal offence. The practice has ruined more lives than the practice of criminal abortion, and ought to be as heavily punished.

